# Identification of two molecular subtypes in canine mast cell tumours through gene expression profiling

**DOI:** 10.1371/journal.pone.0217343

**Published:** 2019-06-19

**Authors:** Lidia H. Pulz, Camila N. Barra, Pamela A. Alexandre, Greice C. Huete, Karine G. Cadrobbi, Adriana T. Nishiya, Silvio Henrique de Freitas, Heidge Fukumasu, Ricardo F. Strefezzi

**Affiliations:** 1 Department of Veterinary Medicine, Faculty of Animal Science and Food Engineering, University of São Paulo, Pirassununga, SP, Brazil; 2 Department of Pathology, Faculty of Veterinary Medicine and Animal Science- FMVZ, University of Sao Paulo, São Paulo, SP, Brazil; 3 Veterinary Hospital Anhembi Morumbi, R. Conselheiro Lafaiete, São Paulo—SP, Anhembi Morumbi University, São Paulo, SP, Brazil; Boston University Henry M Goldman School of Dental Medicine, UNITED STATES

## Abstract

Mast cell tumours (MCTs) are common neoplasms in dogs and are usually regarded as potentially malignant. Several studies have attempted to identify biomarkers to better predict biological behaviours for this tumour. The aim of this study was to identify pathways connected to clinical and histopathological malignancies, shorter survival times, and poor prognoses associated with MCTs. We performed genome-wide gene expression analyses on tissues obtained from 15 dogs with single MCTs, and identified two distinct tumour subtypes—high-risk and low-risk—associated with differences in histological grades, survival times, Ki67 indices, and occurrence of death due the disease. Comparative analyses of RNA sequence profiles revealed 71 genes that were differentially expressed between high- and low-risk MCTs. In addition to these analyses, we also examined gene co-expression networks to explore the biological functions of the identified genes. The network construction revealed 63 gene modules, of which 4 were significantly associated with the more aggressive tumour group. Two of the gene modules positively correlated with high-risk MCTs were also associated with cell proliferation and extracellular matrix-related terms. At the top of the extracellular matrix module category, genes with functions directly related to those of cancer-associated fibroblasts (CAFs) were identified. Immunohistochemical analyses also revealed a greater number of CAFs in high-risk MCTs. This study provides a method for the molecular characterisation of canine MCTs into two distinct subtypes. Our data indicate that proliferation pathways are significantly involved in malignant tumour behaviours, which are known to be relevant for the induction and maintenance of MCTs. Finally, animals presenting high-risk MCTs overexpress genes associated with the extracellular matrix that can be robustly linked to CAF functions. We suggest that CAFs in the MCT stroma contribute to cancer progression.

## Introduction

Canine mast cell tumours (MCTs) are malignant neoplasms composed of atypical mast cells that are characterised by high infiltration capacity and metastatic potential [1, 2]. It is one of the most commonly diagnosed neoplasms in dogs, accounting for 16–21% of cutaneous tumours [3–7]. Since the biological behaviours of MCTs are highly variable, a better understanding of the development and progression, as well as the identification of new prognostic indicators, can help in treating the diseased animals [8].

Like most tumours in animals, canine MCTs are classified based on their histological appearances, which presumably reflect degree of cell differentiation. Besides this classification, prediction of biological behaviours in MCTs could be complemented by additional methods based on histochemical and immunohistochemical prognostic markers [9–12]. However, as in human neoplasms, abundant evidence suggests the presence of unrecognised, relevant subclasses of tumours with respect to their underlying molecular phenotypes and prognoses [13]. Clinically, it is also apparent that histologically identical tumours can behave very differently [14].

The transcriptomes of canine cancers have been investigated mainly by cDNA microarrays in mammary tumours [15, 16], osteosarcomas [17, 18, 19], hemangiosarcomas [20, 21], lymphomas [22, 23], histiocytic sarcomas [24], mast cell tumours [25, 26], and melanoma cell lines [27]. Compared to microarrays, next generation RNA sequencing (RNA-seq) is a more powerful technique, allowing the investigation of gene expression data at a whole-transcriptome level with unprecedented sensitivity and accuracy [28]. Recent investigations of canine tumours with RNA-seq have been carried out in B-cell lymphomas [29], mammary carcinomas [30], squamous cell carcinomas of the head and neck [31], bladder cancers [32], and hemangiosarcomas [33].

In this study, we aimed to identify molecular pathways associated with MCT behaviour using differential expression and co-expression network analyses with RNA-seq-based transcriptomics data from canine MCTs samples. To the best of our knowledge, this is the first study to characterise MCTs using network methods.

## Materials and methods

### Canine tissue samples

Since only animals with single lesions were included in this study, a total of 15 cutaneous MCTs from 15 dogs were utilised. Fresh-frozen and formalin-fixed paraffin-embedded (FFPE) tissue sections of spontaneous canine MCTs were obtained from the Veterinary Hospitals of the School of Veterinary Medicine and Animal Science of the University of São Paulo, Methodist University of São Paulo and the Veterinary Clinic Provet. Samples were acquired from routine cases that were treated by wide surgical resectioning. All experiments were approved by “Ethics Committee for the use of animals” of the School of Veterinary Medicine and Animal Science of the University of São Paulo #2893/2013 and consent for the use of foreskin tissue was provided by the legal guardians of all tissue donors in this study.

All tumours were chosen based on the following inclusion criteria: 1) confirmed histological diagnosis of canine cutaneous MCT; 2) treatment with surgical excision without neoadjuvant chemotherapy (no radiation or chemotherapy before or at the time of tumour removal); 3) availability of follow-up data.

Each tumour was evaluated according to a two-tier grading system into low-grade (grade 1) or high-grade (grade 2) of malignancy [34] by the same veterinary pathologist (R. F. Strefezzi), who was not supplied with any information about the cases. The choice of this histopathological grading criteria eliminates the ambiguity of intermediate grade MCTs and is more accurate in predicting the biological behaviour of this tumour [9, 34].

Follow-up data collection began from the day of first contact, and continued for at least 180 days post-surgery and included details of age, sex, breed, location of the lesions, time to relapse, overall survival and *causa mortis*, when applicable. Overall survival was defined as the interval between surgical excision and last follow-up day or death by any cause. At the end of the study, deaths unrelated to MCT were censored. Nine dogs (60%) received adjuvant chemotherapy. The treatment protocols included combinations of vinblastine, prednisone, and lomustine.

### Ki67 immunohistochemical staining

FFPE tissues were sectioned into 5 μm-thick sections, deparaffinised in xylene, rehydrated in graded ethanol, and rinsed in distilled water. Endogenous peroxidases were blocked by incubating sections in 3% hydrogen peroxide for 5 m. Antigen retrieval was performed by incubating the sections in citrate buffer (pH 6.0) in a pressure cooker for 2 m and cooled for 20 m. All slides were rinsed with 0.05 M phosphate buffered saline (PBS, pH 7.6) with 0.01% Tween 20. The slides were subsequently incubated with mouse monoclonal anti-Ki67 primary antibodies (MIB1; Dako Cytomation Carpinteria, CA) at a dilution of 1:50, in a moist chamber at 4 ^o^C for 16 h (overnight). Following this, slides were incubated with secondary antibodies (Code K4068, ADVANCE^TM^ HRP Link, anti-mouse and anti-rabbit secondary antibodies, Dako, Carpinteria, CA) for 25 m and the reaction was amplified with Advance HRP Enzyme Polymer (Code K4068, ADVANCE^TM^ HRP Enzyme, antibodies polymerised with horseradish peroxidase, Dako Cytomation Carpinteria, CA). The reactions were visualised with 3,4-diaminobenzidine (Liquid DAB + Substrate Chromogen System, Dako Cytomation Carpinteria, CA) and counterstained with Mayer’s haematoxylin.

For the negative control, the primary antibody was replaced with a normal mouse IgG at the same concentration as the primary antibody. The basal layer of the epidermis served as an internal positive control for Ki67.

### Ki67 index

Histological images were evaluated distant from the deep and lateral margins of the tumour mass. For each lesion, a total of five high power fields (HPF) (400x magnification) were selected from areas with the highest percentage of labelled mast cells (“hot spots”) at low magnification (100x magnification). To determine the percentage of proliferating cells (Ki67 index), we counted the number of mast cells showing positive and negative immunostaining for Ki67 in the chosen fields per captured image using the ImageJ software; a minimum of 200 cells for each section were counted. We determined the average percentage of positive mast cells in five fields without prior knowledge of the clinical outcome.

### Definition of high-risk and low-risk MCTs

We used four criteria to divide MCT samples into low- or high-risk groups: histological grade, survival time, Ki67 index and death due the disease. The tumours were graded histologically with a two-tier system (low-grade or grade 1 and high-grade or grade 2). Each parameter received a score, which was added to the histological grade to obtain a combined score or score of malignancy.

For survival time, a score of 1 was designated to animals that remained alive for > 365 days; a score of 2, to those surviving for 180 ≥ 365 days; and a score of 3, for animals with survival times of < 180 days.

Scores to evaluate the Ki67 indices of MCT samples were assigned as follows: a score of 1 was assigned to lesions that showed less than 3% immunoreactive cells; a score of 2 was assigned to lesions that showed 3% to 7% immunoreactive cells; and a score of 3 was assigned to lesions that displayed more than 7% of Ki67-positive cells [35].

Finally, one of the most important biological characteristics to be considered in the study of cancers is their capacity to cause death of the patient. A score to quantify occurrence of death in the studied animals was created by assigning a grade of 0 to all animals that remained alive at the end of the study period, as well as to all censored animals, whereas a score of 3 was assigned to dogs that died due the tumour.

Lesions with a total score of 5 or below were classified as “low-risk” MCTs (samples S02, S03, S05, S06, S07, S08, S09, S11, S13, S14 and S15) and those with a score of 6 or more were defined as “high-risk” MCTs (samples S01, S04, S10 and S12).

### Immunohistochemical staining for αSMA

We evaluated the expression of αSMA in stromal fibroblasts of 44 canine MCTs using immunohistochemistry in FFPE tissues, with a mouse monoclonal antibody (Clone HHF35; Dako Denmark A/S, Glostrup, Denmark). For negative controls, the primary antibody was replaced with normal rabbit or normal mouse IgG at the same concentration as the primary antibody.

All animals from which lesion samples were obtained for the study met the following criteria: 1) all were treated with extensive surgery; 2) none were subjected to neoadjuvant chemotherapy; and 3) follow-up data for at least 180 days was available. Lesions were obtained from routine cases and confirmed as MCTs by histopathology. The Veterinary Hospitals of the School of Veterinary Medicine and Animal Science of the University of São Paulo, Methodist University of São Paulo and the Veterinary Clinic Provet submitted the samples for the analyses.

The same criteria applied for the MCTs analysed by RNA-seq were used to divide the tumours subjected to immunohistochemical analysis into low- and high-risk groups: histological grade, survival time, Ki67 index and death due to disease. The term “low-risk” was attributed to cases with a score lower than 6 and high-risk for tumours with a total score equal to or greater than 6.

The number of positive fibroblasts were counted in five HPFs (400x magnification), using the ImageJ software. Fields were selected from areas with the highest percentage of labelled cells (“hot spots”) at low magnification (100x magnification); the score was calculated from the sum of cells counted in five “hot spots” fields.

### Statistical analysis

Ki67 index values of 44 MCT samples divided into high- and low-risk groups were compared using the Mann-Whitney U test. The number of fibroblasts testing positive for αSMA immunostaining was also compared between the low- and high-risk groups using the Mann-Whitney U test. The data were analysed with GraphPad Prism (version 4.02 for Windows, GraphPad Software, GraphPad Software Inc.) with the significance level set at 5%.

### RNA extraction

Total RNA from each of the 15 fresh-frozen tissue samples were extracted using the RNeasy Mini Kit (Cat No./ID: 74104, Qiagen, Crawley, West Sussex, UK). All samples satisfied the criteria of a nucleic acid 260/280 ratio of approximately 2.0 and were subjected to further analyses. RNA quality was assessed using capillary gel electrophoresis on a BioAnalyzer system (Agilent Technologies Inc., Santa Clara, CA) with RNA 6000 Nano Labchips (Agilent Technologies Inc., Santa Clara, CA, EUA) according to the manufacturer’s instructions. Only samples exhibiting minimal degradation as evidenced by RNA Integrity Number (RIN) ≥ 7.0 were used.

### RNA-seq data analysis

Sequencing was conducted using the Illumina platform (Illumina Inc., San Diego, CA), following the protocols provided by the manufacturer. Total RNA from 15 samples were converted into Illumina sequencing libraries using the TruSeq RNA Sample Preparation Kit in accordance with TruSeq RNA Sample Preparation v2 Guide (Illumina, USA, 2012, Part # 15026495 Rev. D). PolyA RNA was enriched from 1 μg of total RNA using oligo dT-coated magnetic beads, following which, the enriched RNA was fragmented and used for cDNA synthesis. The cDNA was fragmented, blunt-ended, ligated to bar-coded adaptors, and amplified using 15 cycles of PCR. Final library size distribution was validated through quantitative polymerase chain reaction (qPCR) using an Agilent 2100 Bioanalyzer with a KAPA Library Quantification kit (KAPA Biosystems, Foster City, USA). Adapter-ligated cDNA fragment libraries were run on Illumina HiSeq 2500 equipment using the TruSeq PE Cluster Kit and the TruSeq SBS Kit (2x100 bp). An average of 28.4 million PE 100-bp reads were sequenced per sample.

Sequencing quality was evaluated using the FastQC software (http://www.bioinformatics.babraham.ac.uk/projects/fastqc/) and due to its high quality, no filter was applied prior to alignment. Read pairs were aligned to the dog reference genome (CanFam3.1) with *TopHat2/Bowtie2* (tophat.cbcb.umd.edu), allowing two mismatches per read. The sequence alignment map (SAM) files were filtered using Samtools [36] to remove secondary alignments, PCR duplicates, and low-quality alignments. Following this, read counts for each gene was estimated using HTSeq [37]. Gene expression was estimated as counts per million (CPM) and genes which presented at least 1 CPM in at least 4 samples were retained for DE analysis.

DE analysis was performed using the EdgeR package, which is based on negative binomial distributions, in the R environment [38]. Only transcripts with adjusted P-values (*P*_*aj*_) ≤ 0.05 e logFC ≥ |2| were considered to be differentially expressed. To ensure that the differentially expressed genes were sufficient to differentiate between the 2 groups, a hierarchical clustering analysis was performed. Over-/under-expressed genes in canine MCTs were identified on a heatmap.

### Co-expression analysis

Co-expression analysis was used to generate a transcriptional network to investigate associations between gene modules and malignancy grades of MCTs. The analysis was performed using the WGCNA (Weighted Correlation Network Analysis) package in the R environment [39]. From the total of 13,948 genes which passed quality control, only the 6000 most-connected genes were selected due to computational limitations; furthermore, genes with low connectivity were not considered since they are likely to contribute little to the network [40].

Connectivity was calculated as the sum of correlations between one gene and all other genes in the network. To identify modules, a matrix was first generated by calculating Pearson’s correlation coefficients between all genes and raising it to a power β (soft threshold) of 11. Following this, a topological overlap measure (TOM) was calculated by assigning values between 0 and 1 to each pair of genes based on the number of shared neighbours; this was then used to generate a clustering tree whose branches were identified for cutting using the dynamic tree-cutting algorithm [41]. Modules containing a minimum of 30 genes were detected and assigned to colour names. Pearson’s correlation coefficients were used again to measure correlations between modules’ expression profiles (module eigengenes) and the malignancy scores described earlier. Modules with significant correlations (*p* < 0.1) were considered biologically relevant and used for further analysis.

### Functional enrichment analysis

A web-server interactive software tool, GOrilla, was used to identify GO terms enriched in both the differentially expressed genes and co-expression modules associated with tumour malignancy; this was done using a hypergeometric distribution with corrections for false positive rate or false discovery rate (FDR). Genes were converted to their human orthologues to obtain the best functional detail available. Only expressed genes were used as background and terms were considered significant only if *P*_*aj*_ ≤ 0.05.

## Results

### Clinical and histopathological data and Ki67 index values

A total of 15 dogs diagnosed with MCT were included in this study. The median age at diagnosis was 10 years (mean = 9.7 years, range = 3–15 years) and 41.2% of the patients were male. The most common breeds represented in our sample were Labrador Retrievers (5 dogs), Golden Retrievers (3 dogs) and Pit bulls (2 dogs). The remaining dogs in the sample were a poodle, dachshund, Cocker Spaniel, pug and mongrel. The most frequent locations of the MCTs were in the extremities (40% in the limbs and tail) and trunk (33.3%), followed by the abdomen and inguinal regions (13.3% each). A summary of the descriptions of the canine population is shown in Table 1.

**Table 1:** Summary of clinical and histopathological data with their respective identification number of RNA-seq.

RNA-SEQ ID	BREED	AGE YEARS	GENDER	TUMOUR LOCATION	FOLLOW-UP (DAYS)	STATUS	PATNAIK ET AL. 1984 GRADE SYSTEM	TWO-TIER GRADING SYSTEM^a^	KI67 INDEX
**S01**	Dachshund	10	Male	Limbs and tail	658	CS	3	2	7.95%
**S02**	Labrador	11	Female	Trunk	480	LA	1	1	2.92%
**S03**	Poodle	15	Female	Trunk	466	LA	1	1	2.36%
**S04**	Golden Retriever	7	Female	Limbs and tail	308	DT	2	1	23.41%
**S05**	Pug	9	Female	Limbs and tail	587	LA	2	1	5.28%
**S06**	Pitbull	10	Female	Abdomen	254	LA	2	1	3.65%
**S07**	Labrador	11	Female	Trunk	364	LA	2	1	4.87%
**S08**	Cocker	13	Female	Trunk	283	LA	2	1	1.88%
**S09**	Labrador	9	Male	Limbs and tail	390	LA	2	1	3.8%
**S10**	Labrador	5	Female	Limbs and tail	101	DT	2	2	15.48%
**S11**	Golden Retriever	3	Female	Inguinal	232	LA	2	1	0.30%
**S12**	Labrador	10	Male	Limbs and tail	130	DT	1	1	2.11%
**S13**	Golden Retriever	9	Male	Trunk	191	LA	2	1	5.21%
**S14**	Mixed breed	13	Male	Inguinal	1095	LA	2	1	2.89%
**S15**	Pitbull	11	Male	Abdomen	676	LA	1	1	3.43%

Abbreviations: ID: identification number; LA: live animals; CS: censored (deaths unrelated to MCT); DT: death related to mast cell tumour.

^a^ Kiupel et al. (2011)

Histopathological analyses of the MCTs were performed according to the classification system described by Patnaik et al. (1984) [42] and 4 lesions were defined as grade I (26.7%), 10 as grade II (66.7%) and 1 as grade III (6.7%). The tumours were also graded using a two-tier histological system proposed by Kiupel et al. (2011) [34], and 13 (86.7%) MCTs were classified as low-grade while only 2 (13.3%) were classified as having a high grade of malignancy.

Three dogs (S04, S10 and S12) in our sample died due to MCT aggressiveness, which caused a local recurrence of MCTs with new tumour development after surgical removal or disseminated metastases. The MCTs from all the three animals that died were graded as ‘intermediate’ according to the criteria of Patnaik et al. (1984) [42], and only one was diagnosed with MCT of high grade of malignancy by the two-tier histological system. One dog died due to unrelated causes and the remaining 12 cases were alive at the end of the study. The minimum follow-up period post-surgery was 190 days (median = 296 days, range = 101–1095 days) (Table 1).

The Ki67 index was determined by the percentage of neoplastic mast cells exhibiting a positive nuclear signal to anti-Ki67 antibodies. Ki67 scores ranged from 0.30–23.41% (mean = 5.7 ± 5.84%). The Ki67 scores for MCTs from the dogs that died due to tumour aggressiveness were 23.4%, 15.5% and 2.11%. (Table 1). The figures representing the different Ki67 scores are in the Supporting information (S1 Fig).

### Classification of samples as high- or low-risk MCTs

The categorisation of malignancy in the MCTs was based on clinical and histopathological features. We established individual scores for measures of survival time, proliferation index, and death due the disease. Each of these scores was added to the histological grade assigned to obtain a score of malignancy (Table 2). This numerical scale allowed us to divide all MCT cases into two groups: higher scores indicated high risk of malignancy in tumours (samples S01, S04, S10 and S12), whereas lower scores indicated low risk of malignancy (samples S02, S03, S05, S06, S07, S08, S09, S11, S13, S14 and S15).

**Table 2:** Scoring of mast cell tumours.

RNA-SEQ ID	TWO-TIER GRADING SYSTEM ^a^	FOLLOW-UP SCORE	STATUS SCORE	KI67 SCORE	MALIGNANCY SCORE
**S01**	2	1	0	3	6^b^
**S02**	1	1	0	1	3
**S03**	1	1	0	1	3
**S04**	1	2	3	3	9^b^
**S05**	1	1	0	2	4
**S06**	1	2	0	2	5
**S07**	1	2	0	2	5
**S08**	1	2	0	1	4
**S09**	1	1	0	2	4
**S10**	2	3	3	3	11^b^
**S11**	1	2	0	1	4
**S12**	1	3	3	1	8^b^
**S13**	1	2	0	2	5
**S14**	1	1	0	1	3
**S15**	1	1	0	2	4

Assignment of a score for each characteristic considered: histological grade, survival time, Ki67 index and death due the disease. The mixed score (malignancy score) method was determined from these four characteristics, which together gave a numeric value for each tumour.

Abbreviations: ID: identification number

^a^Kiupel et al. (2011)

^b^Malignancy Score ≥ 6 = high-risk MCT

### Canine mast cell tumour transcriptome

The number of paired end (PE) reads sequenced per sample ranged from 22,885,802 to 32,305,518 (an average of 28,404,278 PE reads were generated per sample). These sequences were mapped to the canine reference genome (canFam3.1) with a mean alignment rate of 83%, with a minimum alignment of 77.7% and maximum alignment of 86.4%. On average, the analyses revealed multiple alignments in 11% of multiple and discordant alignments in 2% of the reads. The remaining 79% of the reads were concordant alignments. Data per sample can be seen in Supporting Information (S1 Table).

### Differentially expressed genes in high- and low-risk MCTs

**Fig 1:**
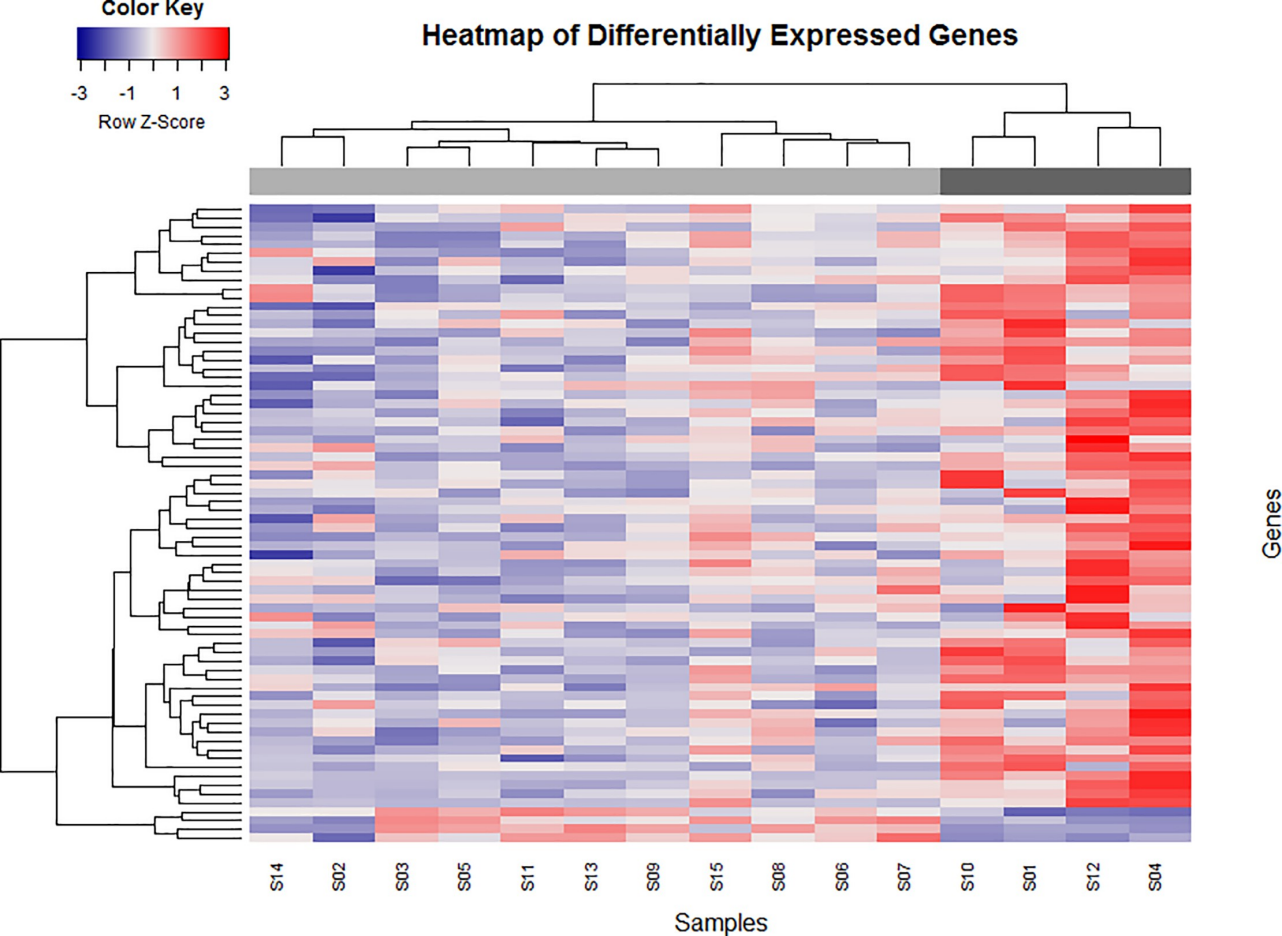
Hierarchical clustering of 15 dog samples based on a subset of 71 differentially expressed genes. A total of 13,948 genes are represented in a heat map. Colour intensity was normalised to log10 (fragments per kilobase of transcript per million mapped reads + 1). Increasing red intensity indicates increased gene expression and increasing blue intensity indicates decreased gene expression, as shown in the scale bar. Statistically significant differences in gene expression (*p* < 0.05) define two molecular subtypes of canine MCTs—high-risk (n = 4) and low-risk MCTs (n = 11). According to the differential gene expression profile, samples were clustered separately and arranged from the low-risk group (left/light grey bar) to the high-risk group (right/ dark grey bar).

Comparisons of transcriptome profiles between high- and low-risk MCTs revealed 71 differentially expressed (DE) genes (*P*_*aj*_ ≤ 0.05) (Fig 1). Of these, 68 were upregulated in the high-risk group while 3 genes were downregulated (Fig 1). A list of the differentially expressed genes with their respective descriptions is available in Table 3.

**Table 3:** List of Differentially expressed (DE) genes between the high-risk MCTs and low-risk MCTs.

*GENE NAME*	*DESCRIPTION*	*PValue*	*P_aj_*
*MMP3*	matrix metallopeptidase 3 (stromelysin 1, progelatinase)	1.17E-20	1.63E-16
*CXCL8*	IL8 or C-X-C Motif Chemokine Ligand 8	3.26E-17	2.27E-13
*IL1B*	Interleukin 1 beta	5.95E-16	2.76E-12
*FMO1*	flavin containing monooxygenase 1	4.96E-11	1.73E-07
*CSF3R*	colony stimulating factor 3 receptor	2.02E-09	5.62E-06
*MAB21L1*	mab-21 like 1	3.41E-09	7.93E-06
*CDO1*	cysteine dioxygenase type 1	7.94E-09	1.58E-05
*ensembl code*	-	1.01E-08	1.77E-05
*GTSF1*	gametocyte specific factor 1	1.19E-08	1.85E-05
*WNT5A*	Wnt family member 5ª	1.39E-08	1.94E-05
*NCAM2*	neural cell adhesion molecule 2	2.62E-08	3.33E-05
*IL18BP*	interleukin 18 binding protein	1.86E-07	0.0002
*LOC483397*	interferon-induced transmembrane protein 1	2.89E-07	0.0003
*IL11*	interleukin 11	5.97E-07	0.0006
*IL18RAP*	interleukin 18 receptor accessory protein	6.50E-07	0.0006
*WIF1*	WNT inhibitory factor 1	7.73E-07	0.0006
*MFSD2B*	major facilitator superfamily domain containing 2B	7.75E-07	0.0006
*EXO1*	exonuclease 1	1.54E-06	0.0012
*PI15*	peptidase inhibitor 15	3.13E-06	0.0023
*KMO*	kynurenine 3-monooxygenase	4.09E-06	0.0029
*COL21A1*	collagen type XXI alpha 1 chain	4.45E-06	0.0030
*LOC102155886/ SAA1(human)*	serum amyloid A protein-like	5.79E-06	0.0037
*MYRIP*	myosin VIIA and Rab interacting protein	6.09E-06	0.0037
*LOC476879/ SAA1 (human)*	serum amyloid A protein-like	9.23E-06	0.0054
*SMPDL3A*	sphingomyelin phosphodiesterase acid like 3ª	1.20E-05	0.0067
*ensembl code*	-	1.38E-05	0.0074
*EDNRB*	endothelin receptor type B	1.61E-05	0.0082
*HTR7*	5-hydroxytryptamine receptor 7	1.74E-05	0.0082
*GNAZ*	G protein subunit alpha z	1.76E-05	0.0082
*CHGA*	chromogranin A	1.73E-05	0.0082
*LOC106557449*	alveolar macrophage chemotactic factor-like	2.04E-05	0.0092
*PGF*	placental growth fator	2.25E-05	0.0098
*LOC484867*	docosahexaenoic acid omega-hydroxylase CYP4F3	2.82E-05	0.0119
*DNM3*	dynamin 3	3.10E-05	0.0127
*KLHL41*	kelch like family member 41	3.30E-05	0.0128
*GAB3*	GRB2 associated binding protein 3	3.31E-05	0.0128
*FRMPD4*	FERM and PDZ domain containing 4	3.62E-05	0.0136
*SAMSN1*	SAM domain, SH3 domain and nuclear localization signals 1	4.05E-05	0.0149
*S100A9 (human)*	S100 calcium binding protein A9	4.40E-05	0.0157
*TNFRSF4*	TNF receptor superfamily member 4	4.53E-05	0.0158
*GRIK4*	glutamate ionotropic receptor kainate type subunit 4	4.76E-05	0.0162
*IGKV2-24 (human)*	immunoglobulin kappa variable 2–24	6.52E-05	0.0216
*PNMT*	phenylethanolamine N-methyltransferase	6.86E-05	0.0218
*SIRPA (human)*	signal regulatory protein alpha	6.77E-05	0.0218
*CLEC4E*	C-type lectin domain family 4 member E	8.64E-05	0.0268
*CSF2RA*	colony stimulating factor 2 receptor alpha subunit	9.24E-05	0.0280
*MUC4 (human)*	mucin 4, cell surface associated	9.47E-05	0.0281
*PLAUR*	plasminogen activator, urokinase receptor	0.0001	0.0289
*SPP2*	secreted phosphoprotein 2	0.0001	0.0289
*SLAMF1*	signaling lymphocytic activation molecule family member 1	0.0001	0.0289
*SOCS1*	suppressor of cytokine signaling 1	0.0001	0.0306
*COL11A2*	collagen type XI alpha 2 chain	0.0001	0.0321
*CLEC5A*	C-type lectin domain family 5 member A	0.0001	0.0327
*IL1RN*	interleukin 1 receptor antagonist	0.0001	0.0327
*CTSE*	cathepsin E	0.0001	0.0334
*IL2RB*	interleukin 2 receptor subunit beta	0.0001	0.0356
*ANOS1*	anosmin 1	0.0001	0.0356
*CYP27B1*	cytochrome P450, family 27, subfamily B, polypeptide 1	0.0002	0.0359
*IDO1*	indoleamine 2,3-dioxygenase 1	0.0002	0.0364
*SLC4A8*	solute carrier family 4 member 8	0.0002	0.0365
*HJURP*	Holliday junction recognition protein	0.0002	0.0365
*SOD2*	superoxide dismutase 2, mitochondrial	0.0002	0.0380
*UCHL1*	ubiquitin C-terminal hydrolase L1	0.0002	0.0380
*C20orf96*	chromosome 24 open reading frame, human C20orf96	0.0002	0.0404
*COL27A1 (human)*	collagen type XXVII alpha 1 chain	0.0002	0.0425
*IL22RA2*	interleukin 22 receptor subunit alpha 2	0.0002	0.0426
*DDC*	dopa decarboxylase	0.0002	0.0470
*GPR35*	G-protein coupled receptor 35-like	0.0002	0.0486
*S100A8*	S100 calcium binding protein A8	0.0002	0.0489
*RFX8*	RFX family member 8, lacking RFX DNA binding domain	0.0003	0.0498
*PCSK1N*	proprotein convertase subtilisin/kexin type 1 inhibitor	0.0003	0.0498

Human orthologues were used in ensembl gene ID that not found a respective gene name in canine transcriptome.

### Co-expression analysis

**Fig 2:**
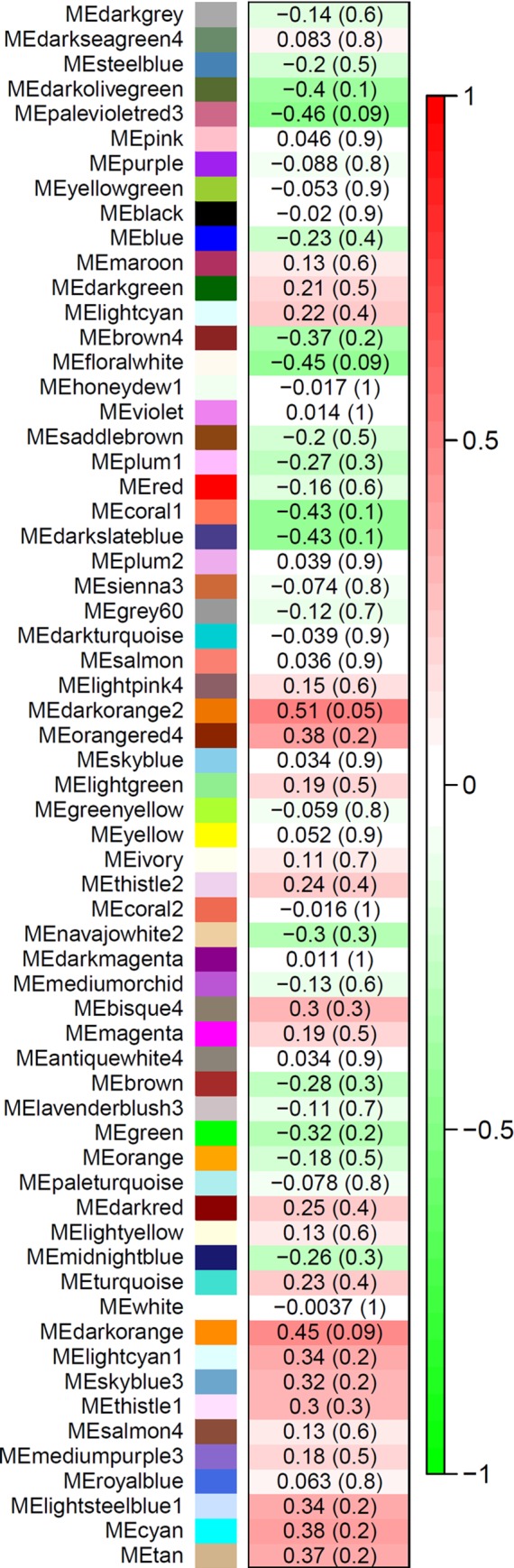
Correlations between module eigengene (ME) values and malignancy scores. Highly connected genes were identified in 63 modules with respective colours considering the 6000 most connected genes in all samples. The level of red intensity indicates degree of positive correlation between module expression and malignancy scores. The level of green intensity indicates the degree of negative correlation of module expression and malignancy scores. The *p*-values for correlation analyses are provided in brackets.

A total of 63 modules were identified when the 6000 most-connected genes from the 15 samples were used for the analysis. Genes that are highly interconnected within the network (modules) are expected to be involved in the same pathways or in roles with related biological functions. The correlation analysis between module eigengene values and malignancy scores resulted in the identification of four significant modules (*P* < 0.1) (Fig 2).

Two modules, *Palevioletred3* (*r* = -0.46, *P* = 0.09) and *Floralwhite* (*r* = -0.45, *P* = 0.09), are negatively correlated with malignancy scores, whereas the other two modules, *Darkorange2* (*r* = 0.51, *P* = 0.05) and *Darkorange* (*r* = 0.45, *P* = 0.09), were positively correlated with malignancy scores (Fig 2). The *Palevioletred3* module included 43 unique genes (S2 Table) in Supporting Information and the *Floralwhite* module included 54 genes (S2 Table). The *Darkorange* module included 85 unique genes (S2 Table) and the *Darkorange2* module with 53 genes (S2 Table) in Supporting Information.

### Functional enrichment analysis

The functional enrichment analysis of all 71 DE genes indicated that these genes were connected to immune/inflammatory responses, mitochondrial activity, and extracellular region (*P*_*aj*_ ≤ 0.05); most of them were found to be up-regulated in the high-risk group. Considering biological processes, there was an enrichment related to response to biotic stimulus (P = 1.28 x 10^−10^, 17/71), chemotaxis (P = 1.37 10^−9^, 12/71), response to lipopolysaccharide (P = 6.77 x 10^−9^, 11/71), immune response (P = 7.33 x 10^−9^, 16/71) and cytokine-mediated signaling pathway (P = 8.32 x 10^−9^, 13/71). When molecular functions were analysed, the enriched gene ontology (GO) terms included oxido-reductase activity (P = 5.97^−11^, 7/71), mono-oxygenase activity (P = 8.26^−9^, 7/71) and G-protein coupled receptor binding (P = 9.61^−7^, 8/71). Interestingly, when cellular components were included in the analysis, genes related to the extracellular region were also enriched (P = 9.65^−10^, 22/71).

A new functional enrichment analysis to test for co-expression was carried out for each significant module. This analysis showed that only those modules positively correlated with MCT risk scores (*Darkorange2* and *Darkorange*) presented significant enrichment in GO (*P*_*aj*_ ≤ 0.05).

**Fig 3:**
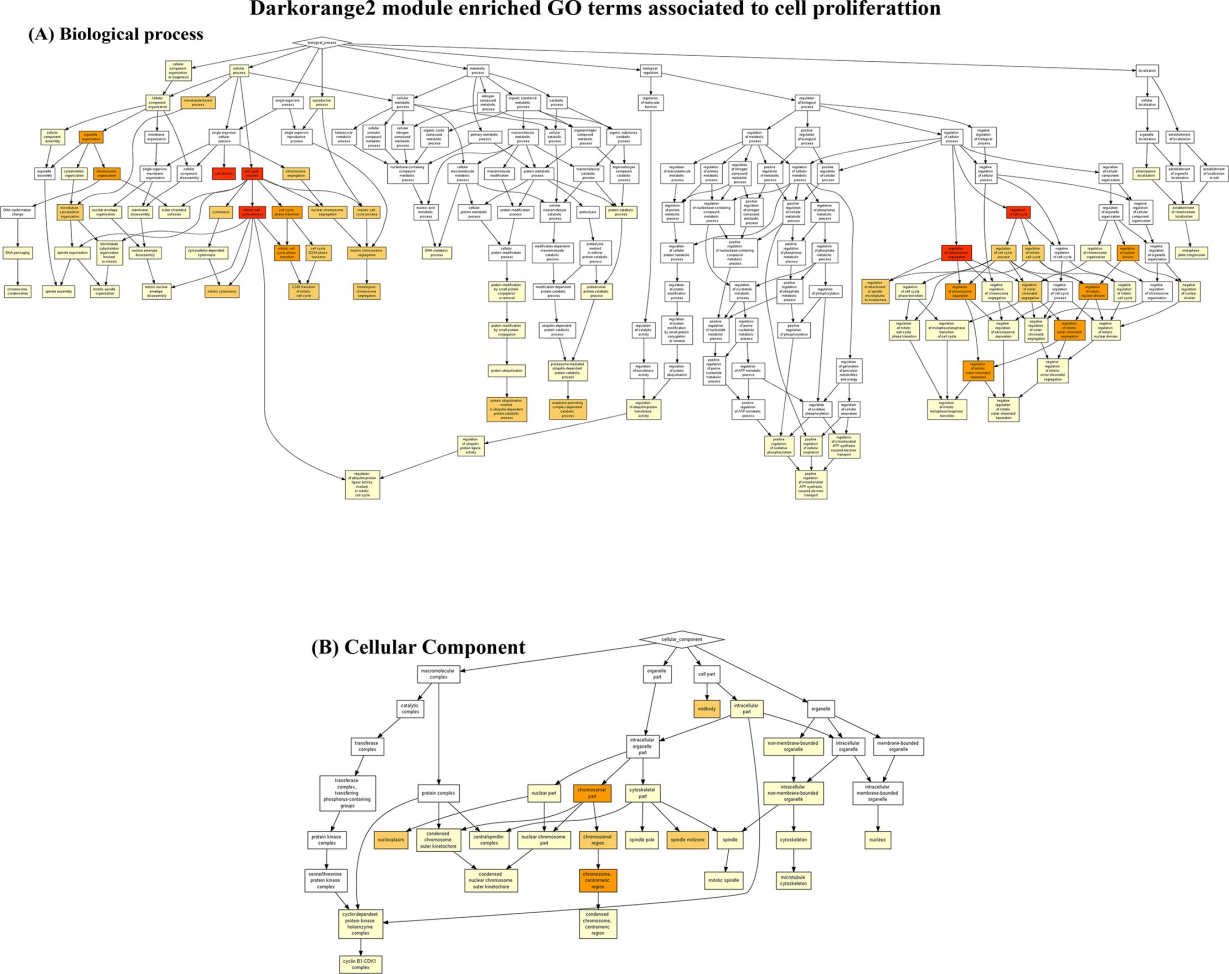
Enriched gene ontology (GO) terms from the *Darkorange2* module associated with cell proliferation. Colour-coded graphical representation reflecting the degree of enrichment of each (A) biological process and (B) cellular components. The higher intensity of red colour represents more significant terms.

The *Darkorange2* module (53 genes) is involved mostly in biological processes related to positive regulation of cell proliferation. The most representative terms in this module included ‘cell cycle process’ (P = 3.08 x 10^−17^), ‘mitotic cell cycle process’ (P = 9.16 x 10^−13^), ‘regulation of chromosome segregation’ (P = 1.18 x 10^−11^) and ‘regulation of cell cycle’ (P = 1.55 x 10^−10^) (Fig 3A). Corroborating this result, the enriched GO terms also included ‘chromosomal part’ (8.1 x 10^−9^), and ‘chromosome, centromeric region’ (5.11 x 10^−8^) (Fig 3B) when cellular components were included in the analysis.

**Fig 4:**
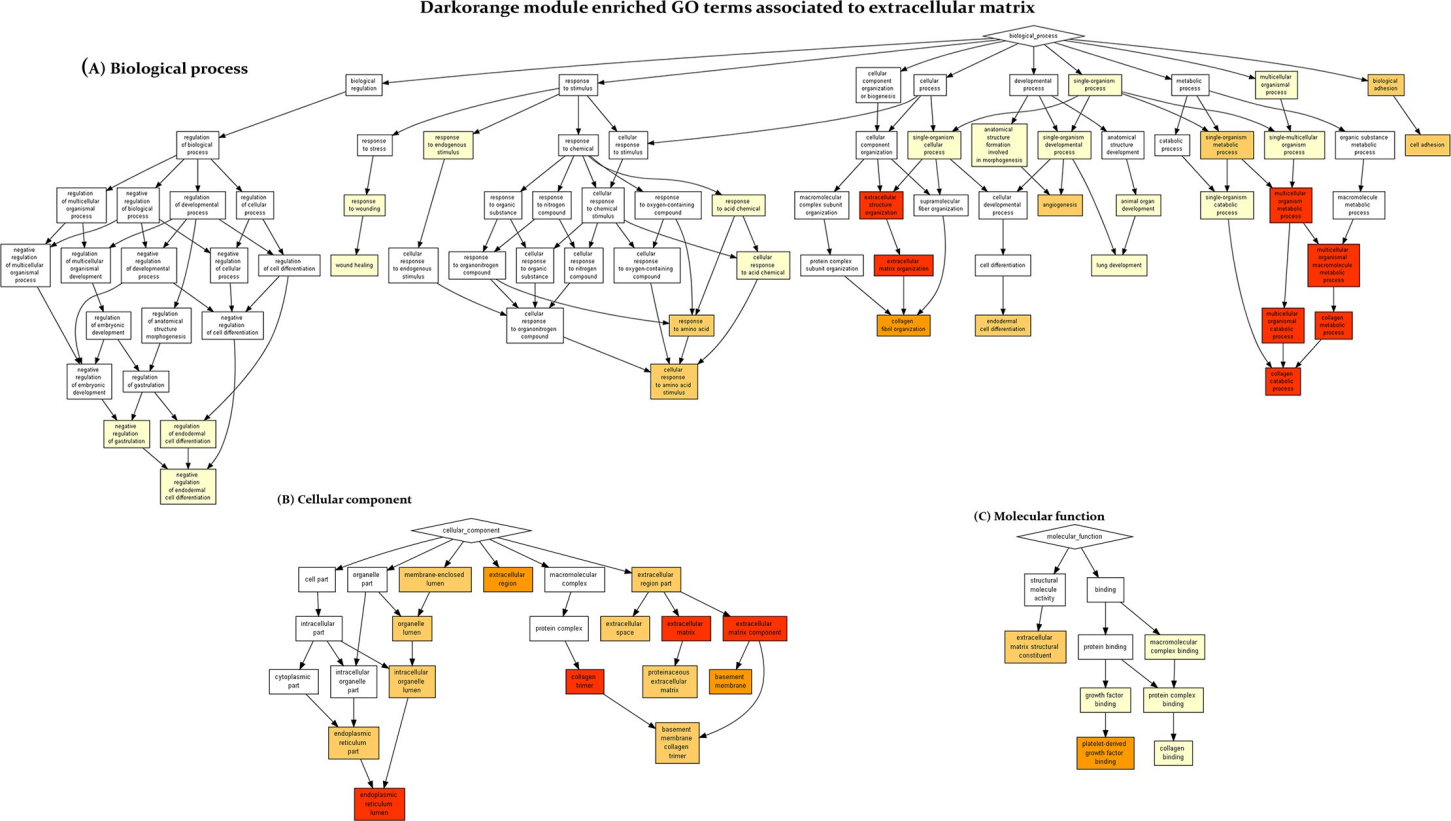
Enriched gene ontology (GO) terms from the *Darkorange* module associated with the extracellular matrix (ECM). Colour-coded graphical representation reflecting the degree of enrichment of (A) biological processes, (B) cellular components and (C) molecular functions. The higher intensity of red colour represents more significant terms.

The second module, *Darkorange* (P = 0.09), showed functional enrichment in genes associated with the extracellular matrix (ECM). The enriched terms were ‘extracellular matrix organisation’ (P = 1.41 x 10^−19^), ‘extracellular structure organisation’ (P = 1.55 x 10^−19^), ‘collagen metabolic process’ (P = 2.28 x 10^−14^), ‘multicellular organismal macromolecule metabolic process’ (P = 4.32 x 10^−14^), and ‘collagen catabolic process’ (P = 1.12 x 10^−13^) (Fig 4A). When cellular components were included in the analysis, enrichment in terms related to ‘extracellular matrix’ (P = 2.59 x 10^−15^), ‘endoplasmic reticulum lumen’ (P = 5.75 x 10^−15^), ‘collagen trimer’ (P = 2.91 x 10^−13^), and ‘extracellular matrix component’ (P = 7.3 x 10^−12^) were also included in the results (Fig 4B). When molecular functions were analysed, the enriched GO terms included ‘platelet-derived growth factor binding’ (P = 5.04 x 10^−8^) and ‘extracellular matrix structural constituent’ (P = 1.02 x 10^−7^) (Fig 4C).

### Biological validation of significant modules

Since the *Darkorange2* module expression profile yielded robust results related to cell proliferation, we proceeded to analyse the Ki67 indices of another set of 44 MCT samples. Comparison of Ki67 indices between low-risk MCTs (samples 01–22, mean = 4.16% ± 0.03%) and high-risk (samples 23–44; mean = 8.91% ± 0.05%) revealed that the percentage of proliferating cells was significantly higher in high-risk MCTs (P = 0.0044) (S2 Fig).

**Fig 5:**
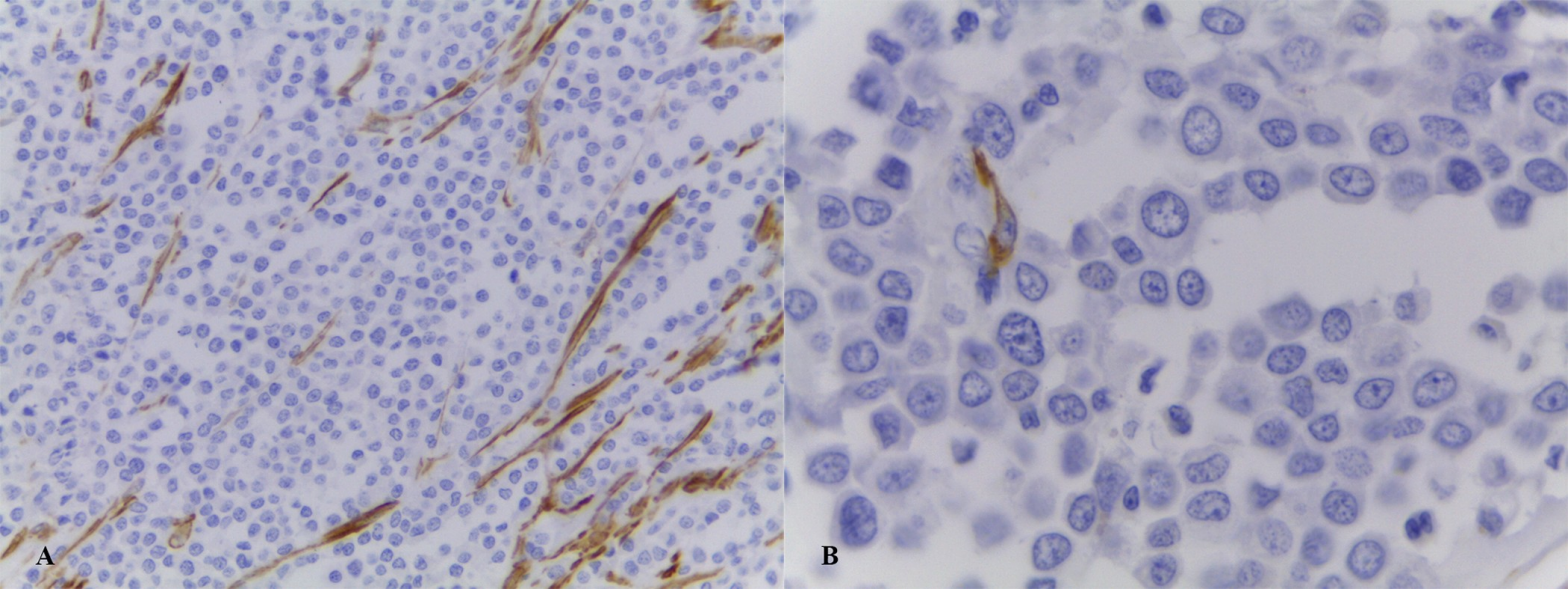
Photomicrographs showing positive immunostaining for α-smooth-muscle actin (αSMA) in canine mast cell tumours (MCTs). (A) Evident stromal fibroblasts immunoexpression (400x magnification). (B) Immunostaining with anti-αSMA antibodies specifically stains the cytoplasm of cancer-associated fibroblasts (CAFs) (1000x magnification). All sections were counterstained with Harris’s haematoxylin.

Since the ‘extracellular matrix module’ (*Darkorange* module) was significantly associated with high-risk MCTs, we further analysed samples to detect the presence of fibroblasts, one of the main components of the stroma that influences the ECM. Fibroblasts within the tumour stroma with a modified phenotype are termed ‘cancer-associated fibroblasts’ (CAFs) and are mostly defined based on the expression of markers such as α-smooth-muscle actin (αSMA). Immunohistochemical analyses to identify and quantify CAFs in 44 canine MCT samples revealed diffuse cytoplasmic immunostaining patterns in fibroblasts (Fig 5).

**Fig 6:**
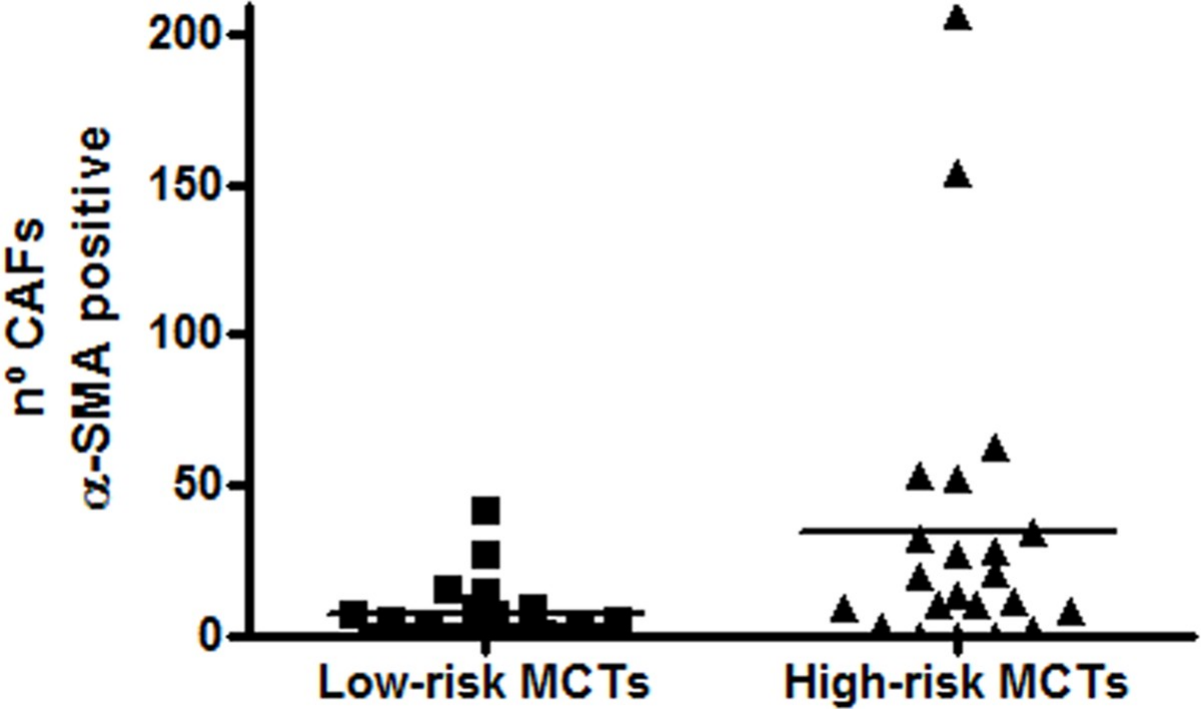
Number of cancer-associated fibroblasts (CAFs) in mast cell tumour stroma of low-risk vs. high-risk MCTs. The number of CAFs in high-risk tumours (22 samples) (P = 0.0021, Mann-Whitney U test) was significantly higher than in low-risk tumours (22 samples).

In the 22 samples that were classified as low-risk (50%), and 22 as high-risk tumours (50%) using the malignancy score (S3 Table), CAFs were present in significantly higher numbers in MCTs classified as high-risk (P = 0.0021) (Fig 6).

## Discussion

In this study, we aimed to identify the mechanisms involved in MCT progression using a transcriptomic approach. Our integrative approach, involving datasets on global gene expression profiles, co-expression analyses, functional enrichment analyses, and validation by immunohistochemistry, revealed that high-risk MCTs are associated with genes that promote increased cellular proliferation and stimuli from the tumour stroma.

First, in a typical genome-wide expression analysis experiment, we used mRNA expression profiles to generate a large number of genes ordered in a ranked list, according to their DE in the high-risk and low-risk MCT groups. The challenge with these data was to extract biological meaning from the list obtained, since single-gene analyses may miss important roles played by whole pathways [43]. Alternatively, we performed co-expression analyses to examine our data at the level of gene sets to obtain information on significant pathways or on ontology.

Since we used proliferation index as a criterion for categorising MCT samples into high- and low-risk groups, our bioinformatics analyses show that a significant number of genes involved in providing proliferation stimuli are upregulated in high-risk MCTs. This result suggests that the molecular pathways identified in our study are biologically relevant to the phenotypes observed. Our results partially corroborate those obtained by Giantin et al. (2014) [25], who demonstrated that a different set of up-regulated genes in undifferentiated MCTs are involved in pathways regulating mitosis. An additional research with the same spontaneous canine cutaneous MCT samples also demonstrated cell cycle gene differentially regulated between aggressive and not aggressive MCTs [26]. More importantly, these results also confirm earlier results that demonstrate the importance of the Ki67 index as a prognostic indicator for MCT, independent of the histopathological grade of the disease [35, 44] and that dogs with high scores of Ki67 expression have shorter survival times [45, 46].

However, the key finding of our study was obtained from the functional enrichment analyses of transcriptomic networks involving the ECM. We must consider that RNA is extracted from the whole MCT tissue. In addition to simply anchoring cells, it is now known that the ECM is an active and complex tissue component [47, 48]. Since fibroblasts are the most abundant cells in connective tissues and are intimately linked to the ECM, both as builders and residents, the modulatory properties of the local ECM are most apparent in fibroblast functions [49].

GO terms associated with the ECM were the most over-represented in the *Darkorange* module, and their expression levels were also positively correlated with malignancy scores. Thus, this module appears to integrate multiple ECM signatures and is likely to play an important role in the progression of MCTs. The *Darkorange* module contains 10 over-expressed collagen genes (S2 Table); as of now, it is not possible to dissociate the role of these molecules from those of the fibroblasts’ matrix microenvironment. In addition, the *Darkorange* module also includes the mRNAs of Lysyl oxidase (LOX) and lysyl oxidase-like-2 (LOXL2), which are matrix enzymes that promote cross-linking of fibrillar collagen and are synthesised by fibroblasts [50]. Stromal LOX expression has been implicated in the development of the metastatic niche [51] and LOXL2 expression is also thought to play a role in promoting invasion [52–55].

Interestingly, another gene that is included in the *“*extracellular matrix module*”* is the platelet derived growth factor receptor-α (PDGFRA), a tyrosine-kinase receptor. Notably, PDGFRA expression on the cell surface is restricted to the stromal compartment in skin and tumour cells [56] and the molecule is reportedly expressed by up to 90% of stromal fibroblasts in solid tumours [57]. Local fibroblasts or fibroblast precursors stimulated by members of the PDGF or transforming growth factor beta (TGF-β) family have generally been considered to be the major source of CAFs [58]. These cells within the tumour stroma contribute structurally and functionally to the progression, growth, and spread of cancers [58–60]. CAFs in the tumour stroma acquire a modified phenotype, similar to wound healing fibroblasts, and are also known as myofibroblasts, reactive stromal fibroblasts, tumour-associated fibroblasts, or activated fibroblasts [58, 61]. Several studies highlight αSMA as an important marker for these cells [62–64].

Based on these findings, we hypothesised that high-risk tumours have an altered microenvironment influenced by CAFs. To confirm this possibility, we demonstrated by immunohistochemistry, that high-risk MCTs have higher number of CAFs in their stroma than low-risk MCTs. The importance of this subpopulation of cells in the tumour microenvironment is emphasised when taken in conjunction with the results of functional enrichment analysis of the *Darkorange* module for angiogenesis. CAFs have been shown to support tumorigenesis by stimulating angiogenesis, cancer cell proliferation, and invasion [56, 65–67].

In accordance with our results, several studies have demonstrated that higher expression levels of αSMA in tumour stromal fibroblasts is an independent prognostic marker for several human cancer types [68–70] and canine neoplasms [71]. In addition, it is interesting to note that there is an association between LOXL2 protein expression and α-SMA-positive stromal fibroblasts in diverse types of solid tumours and that LOXL2 inhibition was efficacious in inhibiting cancer growth in both primary and metastatic xenograft models [72].

## Conclusions

In summary, our study indicates a set of specific genes in MCT samples that have different expression levels in high-risk and low-risk groups. An integrative analytic approach revealed that these genes are related to cellular proliferation and ECM components, mainly from stromal CAFs. While our study provides some insight into the emergent properties of CAFs, more efforts are required to validate and extend our findings.

## Supporting information

S1 Fig Ki67 immunostaining images scores in MCT samples. (A) Ki-67 reactivity in a Score 1 sample, showing less than 3% of immunoreactive cells; (B) Score 2 sample that showed 3% to 7% of specific nuclear staining in mast cells; (C) Score 3 lesion that displayed more than 7% of Ki67-positive cells.
(TIF)

S2 Fig Ki67 index. Comparison of Ki67 indices between low-risk and high-risk MCTs.
(PDF)

S1 Table RNA-seq data. RNA-seq data analyses with the description of each lesion detailing the percentages of alignment to the dog reference genome.
(PDF)

S2 Table Gene co-expression networks. List of component genes of individual modules in each tab: Genes in Palevioletred3 module; Genes in Floralwhite module; Genes in Darkorange2 module; Genes in Darkorange module.
(XLSX)

S3 Table Summary of Clinical, Histopathological and Immunohistochemical data in dogs with MCTs. Breed, age, gender, location of the lesions, follow-up time, survival data, histopathological grades, Ki67 score, quantitative analisys of CAFs with their respective malignancy score.
(PDF)
