# Supplementary figures and images for: Identification of two molecular subtypes in canine mast cell tumours through gene expression profiling
Source: PLoS One. 2019 Jun 19;14(6):e0217343. doi: 10.1371/journal.pone.0217343 (PMC6583995; doi:10.1371/journal.pone.0217343)

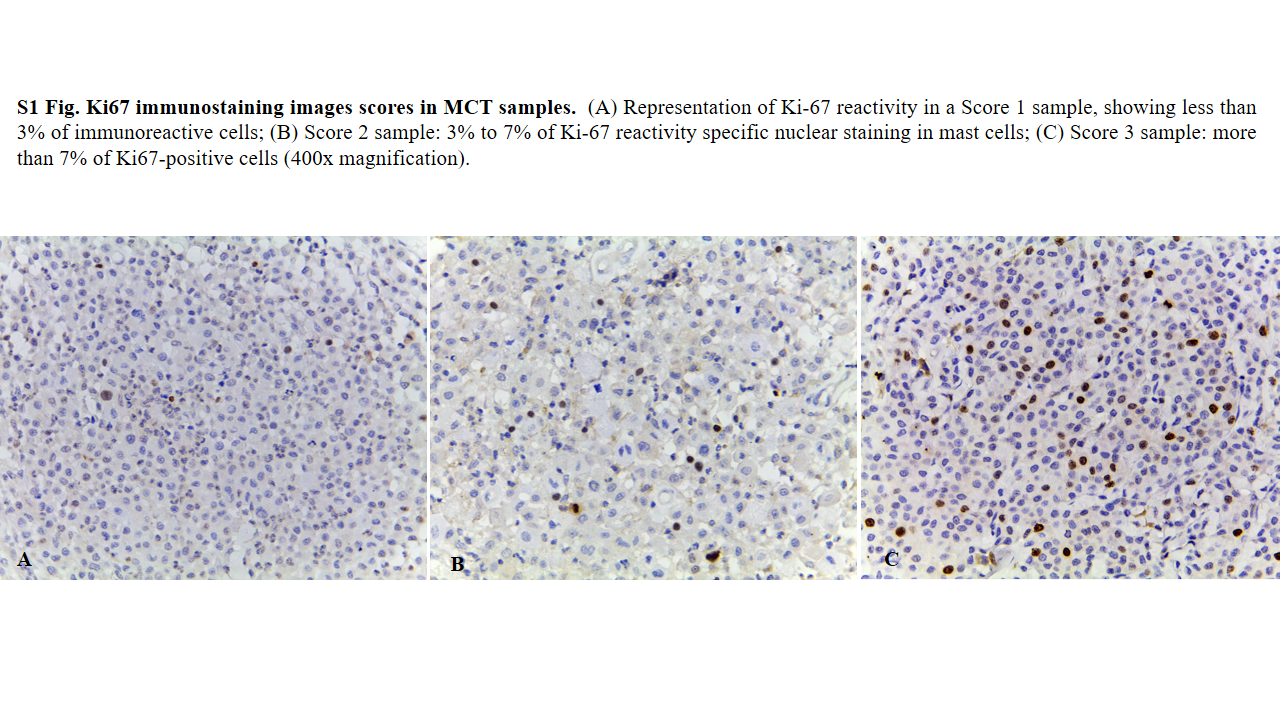

Supplement: S1 Fig — (A) Ki-67 reactivity in a Score 1 sample, showing less than 3% of immunoreactive cells; (B) Score 2 sample that showed 3% to 7% of specific nuclear staining in mast cells; (C) Score 3 lesion that displayed more than 7% of Ki67-positive cells. (TIF) [file pone.0217343.s001.tif]

**S2 Fig. Ki67 index.** Comparison of Ki67 indices between low-risk and high-risk MCTs. (P = 0.0044)

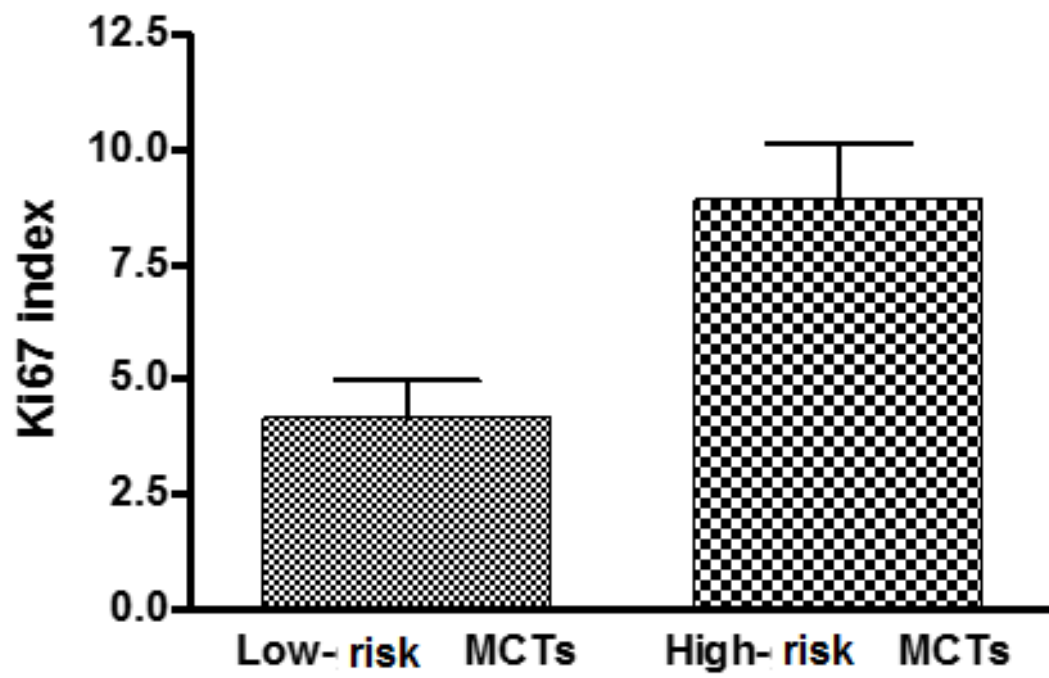

Supplement: S2 Fig — Comparison of Ki67 indices between low-risk and high-risk MCTs. (PDF) [file pone.0217343.s002.pdf]
