# Supplementary material for: Identification of two molecular subtypes in canine mast cell tumours through gene expression profiling
Source: PLoS One. 2019 Jun 19;14(6):e0217343. doi: 10.1371/journal.pone.0217343 (PMC6583995; doi:10.1371/journal.pone.0217343)
Supplement: S1 Table — RNA-seq data analyses with the description of each lesion detailing the percentages of alignment to the dog reference genome. (PDF) [file pone.0217343.s003.pdf]

S1 Table: RNA-seq data analyses with the description of each lesion detailing the percentages of alignment to the dog reference genome

**Samples**	**PE *reads* sequenced**	**Pairs Aligned**	**% Alignment**	**Multiple alignments**	**% Multiple alignments**	**Discordant alignments**	**% Discordant alignments**	**% Concordant alignments**
S01	29190352	22914898	80.5	2829154	12.3	441541	1.9	77.0
S02	27666655	21618809	80.2	2890863	13.4	395265	1.8	76.7
S03	30672031	23212150	77.7	2732208	11.8	340275	1.5	74.6
S04	28642711	22381680	81.8	2351917	10.5	533191	2.4	76.3
S05	29264458	24663416	86.4	2207626	9.0	497104	2.0	82.6
S06	32305518	24637223	78.1	4861085	19.7	483150	2.0	74.8
S07	28226076	23485552	85.3	2991151	12.7	589289	2.5	81.1
S08	29706470	23740961	82.3	3542897	14.9	560530	2.4	78.0
S09	28799480	23616367	84.2	2359155	10.0	549727	2.3	80.1
S10	25683836	21163186	84.7	2146377	10.1	563797	2.7	80.2
S11	29340694	23210833	81.4	2522283	10.9	596017	2.6	77.1
S12	26148434	21603608	85.0	1988881	9.2	584255	2.7	80.4
S13	28758356	23669181	84.8	2388535	10.1	806580	3.4	79.5
S14	22885802	19213272	86.4	1936646	10.1	525150	2.7	81.7
S15	31081917	25783024	85.1	2458563	9.5	551114	2.1	81.2
Mean	28404278	22973137	83.3	2560321	11.1	538073	2.4	79.1

Abbreviations: PE: paired-end
