# Supplementary material for: Identification of two molecular subtypes in canine mast cell tumours through gene expression profiling
Source: PLoS One. 2019 Jun 19;14(6):e0217343. doi: 10.1371/journal.pone.0217343 (PMC6583995; doi:10.1371/journal.pone.0217343)
Supplement: S3 Table — Breed, age, gender, location of the lesions, follow-up time, survival data, histopathological grades, Ki67 score, quantitative analisys of CAFs with their respective malignancy score. (PDF) [file pone.0217343.s005.pdf]

S3 Table. Summary of Clinical, Histopathological and Immunohistochemical data in dogs with MCTs. Breed, age, gender, location of the lesions, follow-up time, survival data, histopathological grades, Ki67 score, quantitative analisys of CAFs with their respective malignancy score.

ID	BREED	AGE (years)	GENDER	TUMOR LOCATION	FOLLOW-UP (days)	STATUS	TWO-TIER GRADING SYSTEM•	Ki67 Index	KI67 SCORE	MALIGNANCY SCORE	CAFs α-SMA POSITIVE (nº)
1	Great Dane	6	male	limbs and tail	840	CS	1	2.13%	1	3	42
2	mixed breed	7	female	limbs and tail	1035	LA	1	1.27%	1	3	0
3	Poodle	7	male	limbs and tail	1450	LA	1	2.66%	1	3	7
4	Boxer	4	female	limbs and tail	1242	CS	1	2.00%	1	3	4
5	Pinscher	7	female	limbs and tail	991	CS	1	0.00%	1	3	5
6	Poodle	15	female	trunk	466	LA	1	2.36%	1	3	9
7	mixed breed	13	male	inguinal	454	LA	1	2.30%	1	3	0
8	Fila	9	male	trunk	1443	CS	1	2.09%	1	3	0
9	Boxer	9	female	trunk	379	CS	1	2.27%	1	3	5
10	mixed breed	9	female	limbs and tail	1286	LA	1	0.00%	1	3	9
11	Poodle	4	female	limbs and tail	2670	LA	1	1.80%	1	3	2
12	mixed breed	5	female	trunk	1500	CS	1	3.09%	2	4	0
13	mixed breed	n.d.	male	inguinal	1800	CS	1	3.97%	2	4	7
14	Fila	9	male	trunk	692	CS	1	6.19%	2	4	27
15	Labrador	9	male	limbs and tail	395	LA	1	3.80%	2	4	0
16	Poodle	7	male	limbs and tail	1898	LA	1	3.93%	2	4	15
17	Poodle	4	female	trunk	2670	LA	1	3.88%	2	4	0
18	Pit bull	12	female	inguinal	303	LA	1	4.80%	2	5	0
19	mixed breed	13	male	limbs and tail	292	LA	1	6.22%	2	5	1
20	mixed breed	5	female	limbs and tail	392	LA	1	8.92%	3	5	0
21	Labrador	3	male	limbs and tail	920	LA	1	15.18%	3	5	14
22	Fila	10	male	trunk	1067	CS	1	12.73%	3	5	0
23	mixed breed	13	female	trunk	224	LA	1	8.07%	3	6	13
24	Boxer	10	male	limbs and tail	551	CS	2	21.87%	3	6	52
25	Golden retriever	9	female	limbs and tail	289	LA	1	8.75%	3	6	8
26	mixed breed	14	female	head and neck	200	DT	1	2.66%	1	7	9
27	Boxer	8	female	limbs and tail	248	DT	1	2.86%	1	7	10
28	Dachshund	12	male	abdomen	751	DT	2	2.27%	1	7	0
29	Boxer	12	male	trunk	1119	DT	2	4.59%	2	8	3

30	Labrador	10	male	limbs and tail	130	DT	1	2.11%	1	8	28
31	Boxer	14	male	trunk	68	DT	1	1.84%	1	8	34
32	Dachshund	12	male	trunk	131	LA	2	13.00%	3	8	21
33	mixed breed	13	female	limbs and tail	407	DT	2	7.67%	3	9	63
34	Boxer	9	female	abdomen	3	DT	2	1.84%	1	9	53
35	Pug	9	female	trunk	308	DT	2	13.76%	3	10	20
36	mixed breed	13	male	inguinal	207	DT	2	12.89%	3	10	27
37	Doberman	9	male	limbs and tail	112	DT	2	6.59%	2	10	32
38	Dachshund	10	male	limbs and tail	222	DT	2	18.78%	3	10	11
39	Poodle	11	male	trunk	228	DT	2	16.04%	3	10	207
40	Boxer	9	female	abdomen	3	DT	2	7.09%	3	11	154
41	Pit Bull	7	female	trunk	51	DT	2	13.12%	3	11	10
42	Labrador	8	female	trunk	120	DT	2	7.25%	3	11	0
43	Labrador	8	female	trunk	120	DT	2	10.05%	3	11	2
44	Labrador	8	female	trunk	120	DT	2	12.98%	3	11	0

Abbreviations: ID: identification number; CAF: cancer-associated fibroblasts; LA: live animals; CS: censored (deaths unrelated to MCT); DT: death related to mast cell; n.d.: no data. • Kiupel et al. (2011)
